# Supplementary figures and images for: Abscisic Acid and Jasmonate Metabolisms Are Jointly Regulated During Senescence in Roots and Leaves of Populus trichocarpa
Source: Int J Mol Sci. 2020 Mar 17;21(6):2042. doi: 10.3390/ijms21062042 (PMC7139941; doi:10.3390/ijms21062042)

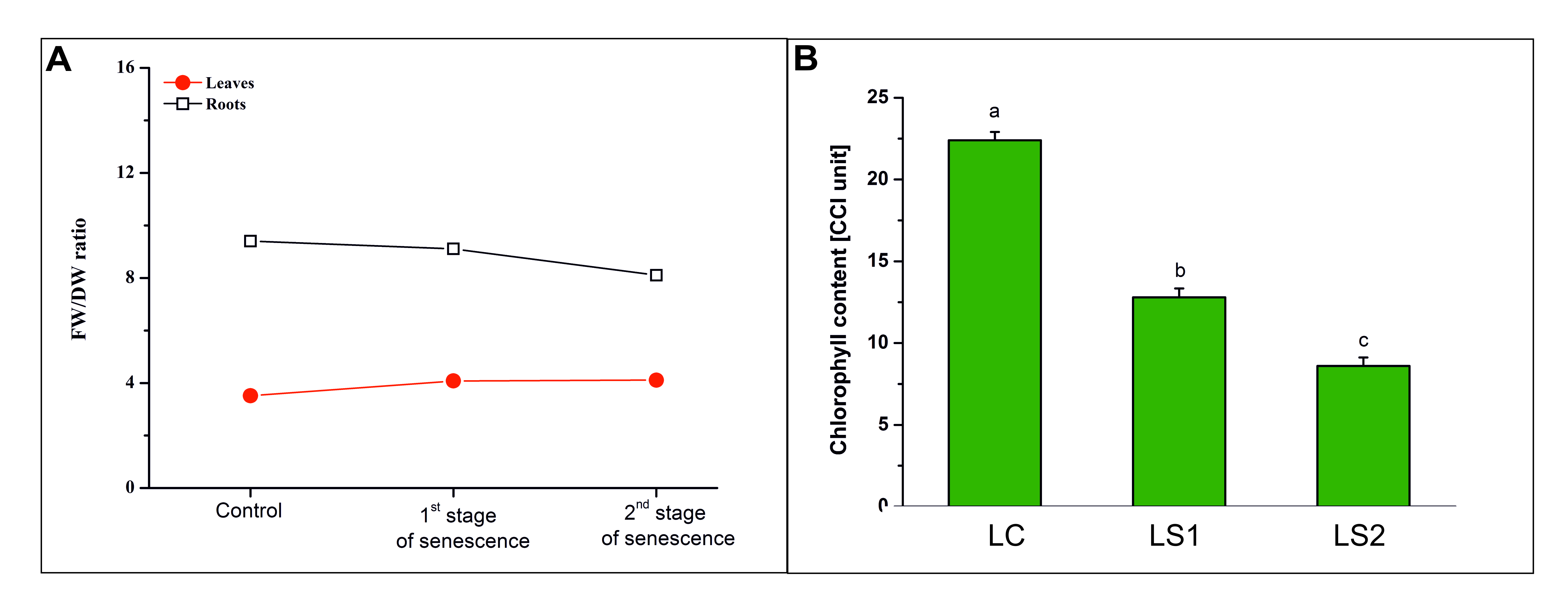

Supplement: Supplementary file 1 [file ijms-21-02042-s001.zip › ijms-717015 supplementary/Supplementary Figure S1.tif]

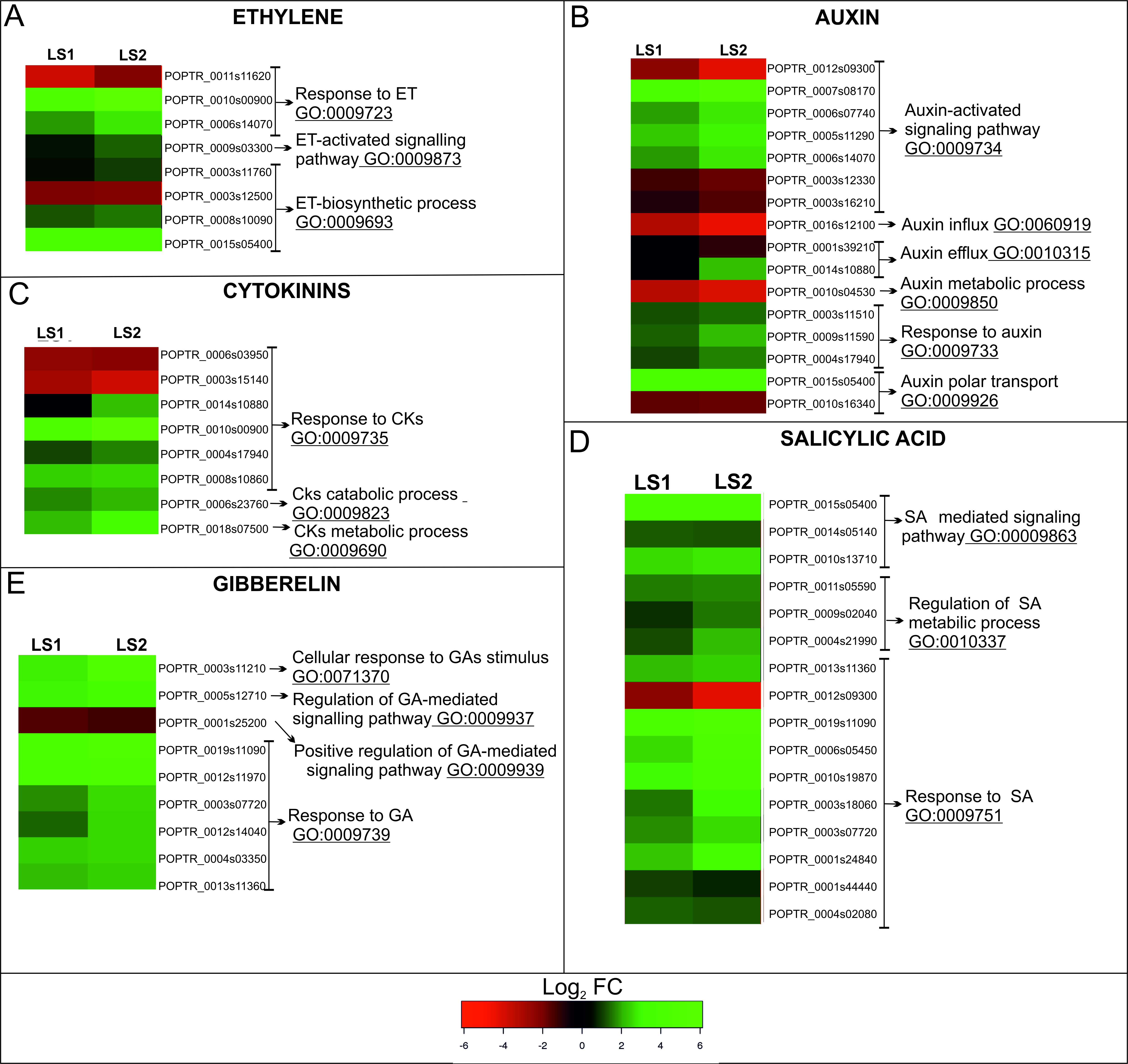

Supplement: Supplementary file 1 [file ijms-21-02042-s001.zip › ijms-717015 supplementary/Supplementary Figure S5.tif]

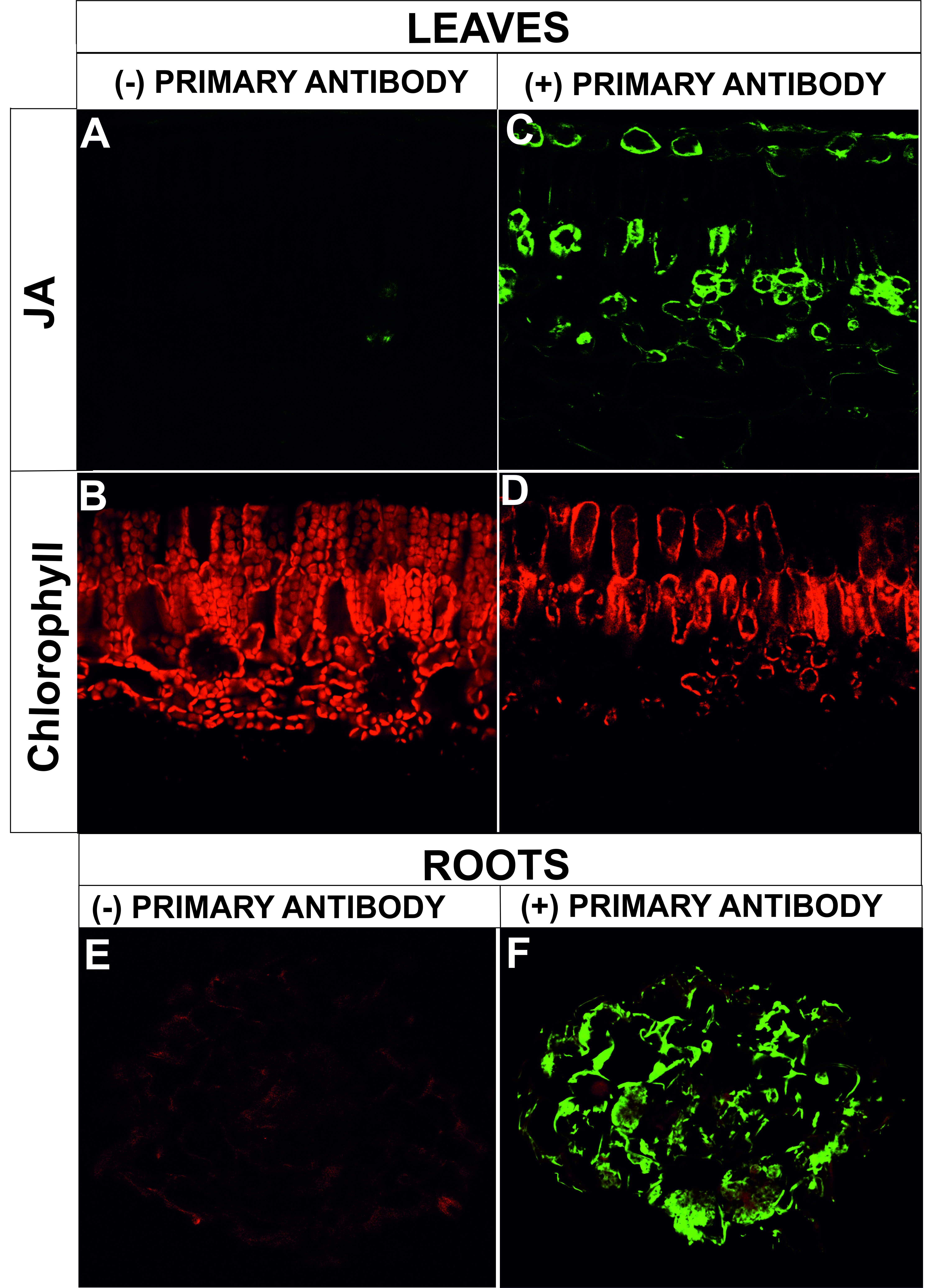

Supplement: Supplementary file 1 [file ijms-21-02042-s001.zip › ijms-717015 supplementary/Supplementary Figure S6.tif]
